# Symptom Trajectories and Attendance in Face-to-Face and Online Psychiatric Follow-Up for Major Depressive Disorder and Anxiety Symptoms: Prospective Observational Cohort Study

**DOI:** 10.2196/97656

**Published:** 2026-10-19

**Authors:** Doğan Akalın, Vesile Şentürk Cankorur

**Affiliations:** 1 Department of Psychiatry Afyonkarahisar State Hospital Afyonkarahisar Türkiye; 2 Department of Psychiatry Faculty of Medicine Ankara University Ankara Türkiye

**Keywords:** telepsychiatry, major depressive disorder, depression, anxiety, treatment adherence

## Abstract

**Background:**

Major depressive disorder is a leading cause of disability, and access to specialist psychiatric follow-up remains limited in many settings. Telepsychiatry may reduce logistical barriers to care, but data on video-based follow-up under routine outpatient conditions in Türkiye are scarce, and comparisons of delivery modalities are frequently limited by baseline imbalance between self-selected groups.

**Objective:**

The aim of the study is to describe symptom trajectories and attendance over 6 months of routine psychiatric follow-up delivered either face-to-face or online and to examine whether outcomes differed between modalities after adjustment for baseline severity.

**Methods:**

In this prospective, nonrandomized observational cohort study, 120 adults with *Diagnostic and Statistical Manual of Mental Disorders, Fifth Edition* (DSM-5) major depressive disorder were followed for 6 months at a university hospital psychiatry department in Ankara, Türkiye (September 2021 to May 2022). Participants entered face-to-face (n=60) or online (n=60) follow-up according to their own preference. Seven sessions were scheduled at approximately 4-week intervals; the Hamilton Depression Rating Scale (HAM-D; primary outcome), the Hamilton Anxiety Rating Scale, and DSM-5 Level 1 and Level 2 measures were administered at baseline and at sessions 1, 2, 4, and 6. Attendance was recorded at all sessions. Between-group comparisons used analysis of covariance adjusted for the corresponding baseline score and for prespecified covariates. Linear mixed models incorporating a group-by-time interaction were fitted to all available observations, and multiple imputation (*m*=20) was performed as a sensitivity analysis.

**Results:**

Depressive symptoms decreased substantially in both groups: mean HAM-D fell from 26.6 to 5.0 in the face-to-face group (81.2% reduction; Cohen *d*=4.86; *P*<.001; n=43) and from 24.1 to 3.3 in the online group (86.3% reduction; Cohen *d*=6.06; *P*<.001; n=49). Response rates were 41 of 43 (95.3%) and 48 of 49 (98%; *P*=.60), and remission rates were 32 of 43 (74.4%) and 43 of 49 (87.8%; *P*=.11). Unadjusted between-group differences at sessions 4 and 6 did not persist after adjustment for baseline severity (session 4: –0.47, 95% CI –1.71 to 0.77; *P*=.46; session 6: –0.79, 95% CI –2.22 to 0.65; *P*=.28), and the group-by-time interaction was not significant (*χ*^2^_3_=1.9; *P*=.59). Anxiety scores did not differ at any time point. Findings were unchanged under multiple imputation, and prescribing patterns were comparable. Attendance across all 7 scheduled sessions was higher in the online group overall (373/420, 88.8% vs 319/420, 76%; *P*<.001), with differences at sessions 2 through 5.

**Conclusions:**

Both modalities were followed by large reductions in depressive symptoms, and between-group differences did not persist after adjustment for baseline severity. The study was not designed as an equivalence trial and cannot establish equivalence. The most consistent difference favoring online follow-up was attendance, although its reasons could not be determined. These findings characterize online psychiatric follow-up as a feasible means of maintaining continuity of routine care rather than as a more effective one.

## Introduction

### Psychiatrist-Led Mental Health Services in Major Depressive and Anxiety Disorders

Major depressive disorder and anxiety disorders are among the most prevalent psychiatric conditions worldwide, representing a substantial burden at both individual and societal levels [1]. These disorders are associated with reduced quality of life, decreased productivity, increased comorbidity with chronic diseases, and growing economic costs for health care systems [2]. A significant proportion of individuals presenting with depressive and anxiety symptoms are referred directly to psychiatrists, placing these specialists at the center of the mental health care delivery system [3].

Mental health professionals play a critical role in the diagnosis, treatment, and follow-up of these conditions; however, access to specialist care remains constrained in many settings. The COVID-19 pandemic further increased demand: an estimated 53.2 million additional cases of major depressive disorder (a 27.6% increase) and 76.2 million additional cases of anxiety disorders occurred globally in 2020, intensifying the need to strengthen mental health systems [4]. Reported barriers to specialist care include shortages of trained professionals, inadequate funding and infrastructure, and the concentration of psychiatric services in urban centers, which leaves large rural populations underserved [5]. In particular, individuals living in rural areas or experiencing transportation difficulties often face substantial obstacles in accessing in-person psychiatric services [6]. Such limitations hinder access to treatment and may negatively affect continuity of care among patients with depressive and anxiety disorders.

Within this context, telepsychiatry, which enables psychiatrists to communicate with patients through real-time video, has been increasingly adopted in routine psychiatric practice. In a nationwide survey of 354 patients with mental disorders in Poland, the proportion preferring video consultation rose from 47% in 2020 to 96% in 2023, and most respondents regarded it as a complementary modality for continuing established treatment rather than as a substitute for initial face-to-face assessment [7]. By providing flexibility in time, location, and resource allocation, telepsychiatry offers a potentially valuable option, particularly in regions where mental health services are limited.

In Türkiye, mental health services are still predominantly hospital-based, while community-oriented and digital mental health practices remain limited. Telepsychiatry has attracted attention mainly over the past decade and was partially implemented in clinical settings during the COVID-19 pandemic. However, the existing Turkish literature is largely theoretical or focused on child and adolescent populations [8,9], and we are not aware of published longitudinal data on psychiatrist-led telepsychiatric follow-up in adults. Describing how face-to-face and online psychiatric follow-up proceed in this setting therefore provides information relevant both to clinical practice and to the integration of digital services into routine psychiatric care.

### Telepsychiatry and Online Interventions in Major Depressive Disorder

Psychiatrist-led telepsychiatry delivered through real-time video has increasingly been described as a viable alternative to traditional face-to-face treatment for major depressive disorder. Systematic reviews focusing on depressive disorders have reported that telepsychiatry reduces depressive symptom severity [10,11], and a network meta-analysis of trials conducted in low- and middle-income countries indicated that telepsychiatric interventions improve access to depression care and produce clinically meaningful symptom reductions [12].

Randomized controlled trials and meta-analyses of such trials have reported that real-time video-delivered psychiatric care produces improvements in depressive symptoms comparable to those achieved face-to-face, with no difference in treatment satisfaction [13,14]. Collectively, these findings suggest that telepsychiatry may represent not merely an alternative delivery modality but a potentially sustainable approach to maintaining access to and continuity of care in patients with major depressive disorder [15].

Despite this evidence, recent reviews have identified methodological limitations within the telepsychiatry literature, including heterogeneity in diagnostic criteria and symptom severity, relatively short follow-up periods, and limited data on longer-term outcomes in patients with major depressive disorder [10,13], including in studies conducted in young populations [16]. In addition, patients enrolled in trials of remotely delivered psychotherapy for depression have been found to differ from those in face-to-face trials, reporting a longer duration of depression and being less likely to have received previous treatment, which limits the generalizability of findings across populations [17]. A further limitation is that most of this evidence derives from randomized trials, in which allocation is controlled and the structured research environment may itself foster adherence; how the 2 modalities compare under routine outpatient conditions, where patients choose their own follow-up modality, has been less well described.

### Objective of the Study

The aim of this study was to describe symptom trajectories, treatment continuity, and attendance over 6 months of routine psychiatric follow-up delivered either face-to-face or online to adults with major depressive disorder and to examine whether outcomes differed between the 2 modalities after adjustment for baseline symptom severity and other potential confounders. Because allocation was determined by patient preference rather than randomization, the study was designed as an observational comparison; it was not intended to establish equivalence between modalities, and the findings are interpreted as associations rather than as causal effects of delivery modality.

## Methods

### Study Design and Setting

This was a prospective, nonrandomized observational cohort study conducted at the Department of Psychiatry, Ankara University Faculty of Medicine, a tertiary university hospital in Ankara, Türkiye. Adults with major depressive disorder were followed for 6 months in either face-to-face or online psychiatric care according to their own preference. Because allocation was based on patient preference rather than randomization, the study was designed and is reported as an observational comparison; findings are interpreted as associations rather than as evidence of a causal effect of delivery modality. Reporting follows the STROBE (Strengthening the Reporting of Observational Studies in Epidemiology) statement for observational studies (Multimedia Appendix 1).

### Participants and Recruitment

Participants were recruited between September 2021 and May 2022 from patients presenting to the adult psychiatry outpatient clinic. The intention was to invite all consecutive patients meeting the eligibility criteria. In practice, eligible patients were identified and referred to the study by the outpatient psychiatrists providing routine care, and some eligible patients may not have been referred; recruitment is therefore best described as consecutive in intention but incomplete in practice. Patients who declined were treated according to routine clinical practice; the number of patients who declined and their reasons were not systematically recorded.

Of 125 patients assessed for eligibility, 5 were excluded because of active suicidal ideation. The remaining 120 provided written informed consent and were enrolled. Each participant selected the follow-up modality themselves, resulting in 60 participants in the face-to-face group and 60 in the online group. The treating clinicians referred patients to the study but did not influence the choice of follow-up modality, and no restrictions were applied to the number of participants entering either group.

Inclusion criteria were age 21 to 65 years, a diagnosis of major depressive disorder established by a psychiatrist according to *Diagnostic and Statistical Manual of Mental Disorders, Fifth Edition* (DSM-5) criteria, treatment on an outpatient basis, and literacy sufficient to complete the study procedures. Participation in the online group additionally required access to the equipment necessary for internet-based videoconferencing. Exclusion criteria were treatment-resistant major depressive disorder, active suicide risk, need for inpatient psychiatric treatment, lack of access to the equipment required for online follow-up, illiteracy, moderate or severe intellectual disability, major neurocognitive disorder, psychotic disorders, neurological disease involving the central nervous system, and the use of addictive substances such as cocaine, cannabis, opioids, or hallucinogens.

In total, 12 of 120 (10%) participants discontinued follow-up entirely, 9 of 60 (15%) in the face-to-face group and 3 of 60 (5%) in the online group. Attendance at individual sessions varied among the remaining participants and is reported for each time point. The inclusion process and participant numbers at each stage are summarized in Figure 1.

**Figure 1:**
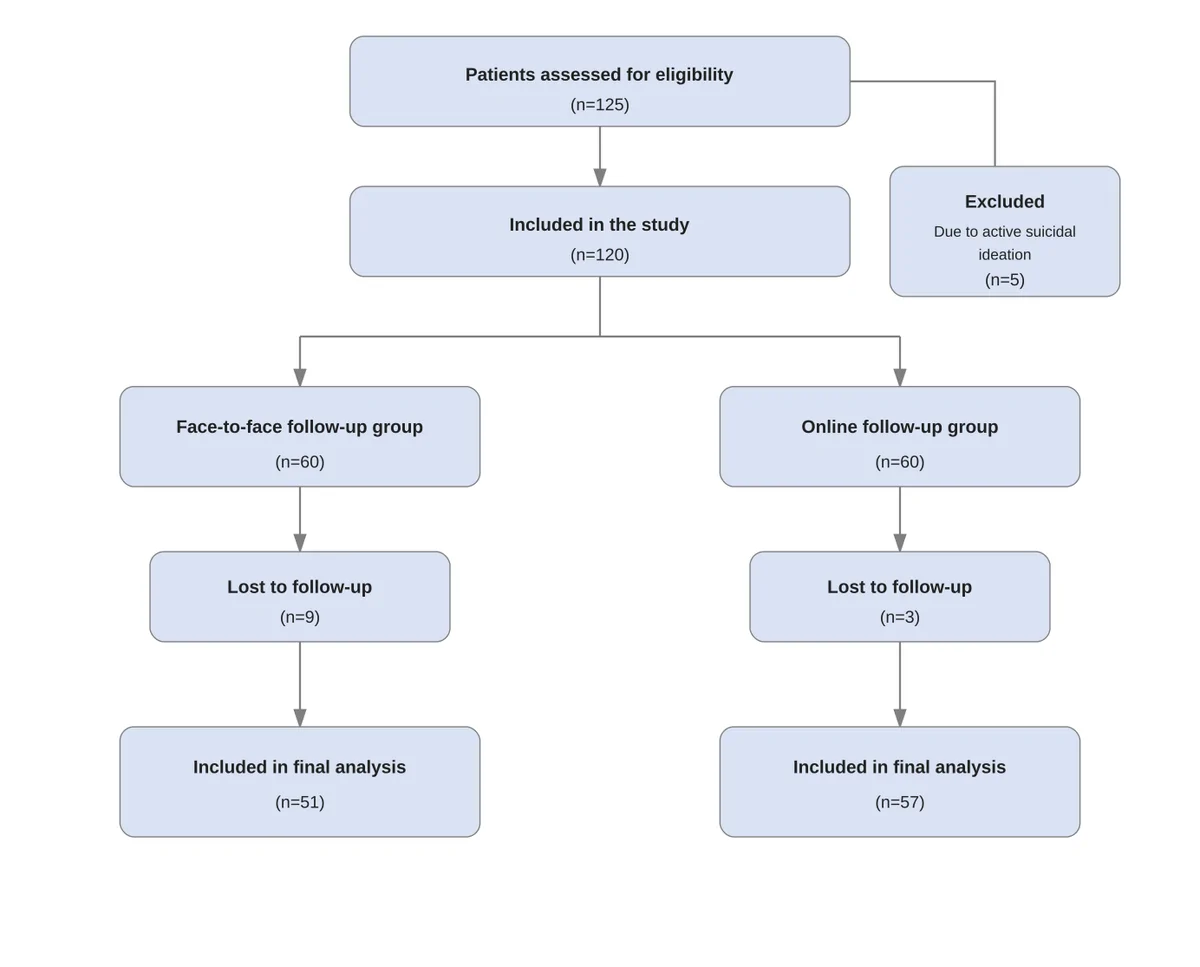
Participant flow diagram following STROBE (Strengthening the Reporting of Observational Studies in Epidemiology) recommendations, illustrating patient screening, exclusion due to active suicidal ideation, allocation to face-to-face or online follow-up based on patient preference, loss to follow-up, and inclusion in the final analyses.

### Ethical Considerations

This study was reviewed and approved by the Ankara University Faculty of Medicine Human Research Ethics Committee (approval İ3-205-21; March 11, 2021). All procedures were conducted in accordance with the Declaration of Helsinki and relevant national research regulations. All participants were fully informed about the purpose, procedures, and voluntary nature of the study and provided written and verbal informed consent before participation. Confidentiality and data privacy were maintained throughout the study, with all participant information anonymized and securely stored. No participant received financial or material compensation for participation.

### Diagnostic Assessment

Psychiatric diagnoses were established by a psychiatrist through clinical evaluation based on DSM-5 diagnostic criteria, including systematic assessment of differential diagnoses and of the exclusion criteria listed earlier. A structured or semistructured diagnostic interview, such as the Structured Clinical Interview for DSM-5 or the Mini International Neuropsychiatric Interview, was not administered, in keeping with routine outpatient practice at the study site. The standardized psychometric instruments described in the Measures section were administered after enrollment to characterize the presence and severity of depressive and anxiety symptoms and served as outcome measures rather than as diagnostic instruments.

### Follow-Up Procedure

After the baseline interview (session 0), 6 follow-up sessions were scheduled at approximately 4-week intervals, giving a total of 7 sessions over 6 months per participant. The interval between the baseline interview and the first follow-up session was therefore approximately 4 weeks. Sessions are numbered throughout this report as session 0 (baseline) and sessions 1 to 6 (follow-up).

Sessions followed a common protocol in both groups (Multimedia Appendix 2). The baseline interview lasted approximately 60 minutes, and follow-up sessions lasted approximately 30 minutes. Each follow-up session comprised assessment of current psychiatric symptoms according to DSM-5 criteria, administration of the scheduled outcome measures, review of treatment adherence and medication-related adverse effects, assessment of daily, interpersonal, and occupational functioning with individualized behavioral recommendations where appropriate, clinical assessment of suicide risk, and psychoeducation covering the nature of depression, the treatment process, relapse risk, medication effects, sleep hygiene, stress management, and the maintenance of regular daily routines. No structured psychotherapy protocol was delivered; sessions reflected routine psychiatric outpatient practice. The timing, structure, and clinical content of sessions were identical in the 2 groups, and the mode of delivery was the only intended difference between them.

Standardized scales were administered at baseline and at sessions 1, 2, 4, and 6. Scales were not administered at sessions 3 and 5, which were conducted as routine clinical follow-up without additional psychometric assessment. This intermittent schedule was predefined in the study protocol in order to reduce repeated assessment burden and to limit disruption of the clinical encounter, while still allowing symptom trajectories to be monitored at regular intervals. The same schedule was applied to both groups. Attendance, by contrast, was recorded at all 7 scheduled sessions as part of routine clinical documentation.

In the face-to-face group, all sessions were conducted in the outpatient clinic under conditions ensuring confidentiality and privacy. The self-report DSM-5 forms were provided in printed format and completed before the session. In the online group, sessions were conducted by video call with the camera on; audio and video quality were checked before each session began. The self-report forms were distributed electronically before the session and returned digitally afterward. In both groups, the Hamilton Depression Rating Scale (HAM-D) and Hamilton Anxiety Rating Scale (HAM-A) were administered by the interviewing psychiatrist at the end of the session.

### Online Platform and Privacy

Online sessions were conducted using Zoom (Zoom Video Communications) or WhatsApp video calls, according to each participant’s preference and technical accessibility. Personal rather than institutional accounts were used, and the platform could vary between sessions for the same participant; the platform used at each session was not systematically recorded. WhatsApp video calls are end-to-end encrypted by default. Zoom applies end-to-end encryption only when this option is explicitly enabled at the account level, and it was not verified whether this setting was active during the study period. Neither platform was covered by an institutional data-processing agreement. Sessions were not recorded. Participants provided written informed consent that specifically covered the use of videoconferencing applications for follow-up sessions and the associated confidentiality considerations. Participants were instructed to attend sessions alone in a private room; at the beginning of each session, the clinician confirmed that the participant was alone and able to speak confidentially, and a reminder regarding privacy and confidentiality was provided.

### Measures

#### Overview

At baseline, all participants completed a sociodemographic and clinical data form and the instruments described in this section. The DSM-5 Level 1 Cross-Cutting Symptom Measure, the DSM-5 Level 2 Depression Scale, the HAM-D, and the HAM-A were readministered at sessions 1, 2, 4, and 6; the Suicide Probability Scale (SPS) was administered at baseline only.

Three measures of depressive symptoms were used because they capture different perspectives on the same construct: the HAM-D provides a clinician-rated assessment of symptom severity, the DSM-5 Level 2 Depression Scale provides a dimensional self-report of depressive symptoms over the preceding week, and the DSM-5 Level 1 measure is a brief screening instrument covering 13 symptom domains that was used to monitor the broader symptom profile rather than to quantify depression severity. The HAM-D total score was designated as the primary outcome; all other measures were treated as secondary or exploratory.

#### DSM-5 Level 1 Cross-Cutting Symptom Measure

Developed by the American Psychiatric Association, this self-report instrument screens 13 psychiatric symptom domains: depression, anger, mania, anxiety, somatic symptoms, suicidal ideation, psychosis, sleep problems, memory, repetitive thoughts and behaviors, dissociation, personality functioning, and substance use. Items are rated from 0 to 4 according to symptom severity during the preceding 2 weeks, and each domain is evaluated against a predefined threshold, indicating the need for further clinical inquiry [18]. The measure is a screening instrument rather than a severity index; domain scores were therefore dichotomized at the recommended thresholds and are reported descriptively. The Turkish version of the adult form was validated by Çökmüş et al [19].

#### DSM-5 Level 2 Depression Scale

Developed by the American Psychiatric Association on the basis of the Patient-Reported Outcomes Measurement Information System (PROMIS), this self-report scale comprises 8 items rated from 1 to 5 and assesses depressive symptoms over the preceding 7 days; raw scores are converted to standardized *T* scores, which were used in all analyses [20]. The Turkish version was validated by Aydın et al [21].

#### HAM-D

The HAM-D is a clinician-administered measure of depressive symptom severity [22]. The 17-item version assesses affective, cognitive, and somatic features and yields a total score ranging from 0 to 52, with higher scores indicating greater severity. Severity bands were defined according to the empirically derived cutoffs of Zimmerman et al [23]: no depression (0-7), mild (8-16), moderate (17-23), and severe (≥24). The Turkish validation was conducted by Akdemir et al [24].

#### HAM-A

The HAM-A is a 14-item clinician-administered measure of anxiety severity [25]. Each item is scored from 0 to 4, producing a total score from 0 to 56. Structured interview guides have been developed to improve the interrater reliability of the scale [26]. The Turkish version was evaluated by Yazıcı et al [27] in a clinical sample using a structured interview guide and was reported to show adequate interrater reliability and validity, with criterion validity assessed against the State-Trait Anxiety Inventory.

#### SPS

The SPS is a 36-item self-report instrument developed by Cull and Gill [28] to assess suicide risk in adolescents and adults. Items are rated on a 4-point scale (1=never or rarely, 2=sometimes, 3=often, and 4=most of the time or always), yielding total scores from 36 to 144, with higher scores indicating greater suicide probability. The instrument comprises 4 subscales: hopelessness, suicidal ideation, negative self-evaluation, and hostility [28]. The Turkish translation used in this study was that of Eskin [29]. In a Turkish clinical sample of psychiatric inpatients and outpatients, confirmatory factor analysis supported the original 4-factor structure, and the scale showed high internal consistency and adequate split-half reliability, with an optimal total-score cutoff of 110 [30]. In that clinical validation study, items 5 and 19 showed low factor loadings and the lowest item-total correlations of the scale; the same 2 items were subsequently found to be problematic in a revised Turkish form, where their removal increased the internal consistency of the dimension containing them [31]. The hopelessness subscale was therefore computed both with and without these 2 items, and both scores are reported. In the present study, subscale scores were used, and the total score was not analyzed.

### Clinicians and Outcome Assessment

All study interviews and clinician-rated assessments (HAM-D and HAM-A) were conducted by a single psychiatrist (DA) in both groups throughout the 6-month follow-up period. Because all assessments were performed by the same rater, treatment modality was not confounded with rater, and interrater reliability was therefore not applicable. The rater could not be blinded to the follow-up modality, as the mode of delivery was inherently apparent during each session.

### Safety Monitoring and Risk Management

Suicide risk was assessed during the eligibility evaluation before enrollment, based on a clinical interview covering suicidal ideation, intent, and plan. Patients reporting active suicidal ideation or judged to be at high risk were not eligible; enrollment was discontinued, and they were referred for further psychiatric evaluation and to the appropriate service for inpatient or outpatient treatment according to the severity and immediacy of the risk. In total, 5 patients were excluded on this basis, and no patient identified as being at high risk entered either study group.

The suicidal ideation item of the DSM-5 Level 1 measure was administered after enrollment as part of the assessment battery and was treated as a screening signal requiring clinical evaluation rather than as a diagnostic indicator. At baseline, 8 (13.3%) participants in the face-to-face group and 13 (21.7%) in the online group scored above the screening threshold; in every case, the score corresponded to the lowest severity level of the item, and clinical assessment confirmed the absence of active suicidal ideation, intent, or plan. This accounts for the difference between the number of patients excluded for suicidality and the proportion screening positive after enrollment.

Suicide risk was reassessed clinically by the interviewing psychiatrist at every follow-up session. A predefined protocol was in place for emergent risk: participants could be evaluated further on the same day, online participants could be converted to an in-person assessment, and referral to the outpatient clinic or to emergency psychiatric services was available according to the level of risk. No participant required such conversion or referral, and no participant was withdrawn during follow-up because of newly emerged active suicidal ideation. The same protocol applied to any clinically significant deterioration identified during follow-up, including worsening of depressive or anxiety symptoms in the absence of suicidal ideation; in such cases, participants could be referred to the outpatient clinic for additional evaluation and treatment adjustment on the same day. Referral for treatment adjustment occurred as part of routine care in both groups.

### Interpretation of Screening Findings

The DSM-5 Level 1 measure was administered after enrollment as part of the assessment battery and was not used for eligibility decisions. Eligibility, including the exclusion of psychotic disorders and bipolar disorder, was determined by clinical evaluation before enrollment. Domain scores above the screening threshold were treated as signals prompting clinical inquiry at the corresponding session rather than as indications of a disorder. Positive screens in the psychosis, mania, and suicidal ideation domains were reviewed clinically by the interviewing psychiatrist, and in no case was a psychotic or manic syndrome identified during follow-up. This distinction is reported here because the proportion of participants screening above threshold in these domains exceeded what would be expected, given the exclusion criteria.

### Pharmacological Treatment

All participants received routine pharmacological treatment. Prescribing decisions were made by the 8 psychiatrists providing routine outpatient care in the same department, independently of the study assessments. Patients were assigned to these clinicians according to the routine outpatient workflow, without regard to follow-up modality, so that the same pool of prescribers served both groups. At follow-up sessions, participants were referred to the outpatient clinic when the interviewing psychiatrist judged that additional clinical evaluation or treatment modification was required; medication was then reviewed and adjusted in accordance with routine clinical practice and current treatment guidelines.

Antidepressant class, active agent, and dose at baseline and at the final session, together with any dose increase, addition of a second agent, augmentation, prescription of a hypnotic, and documented nonadherence during follow-up, were extracted from clinical records and compared between groups. For this purpose, mirtazapine at doses of 30 mg or above was classified as an antidepressant and doses of 15 mg or below as a hypnotic; trazodone was classified as a hypnotic in all cases. Pharmacological treatment followed routine clinical practice rather than a standardized protocol and is therefore reported descriptively.

### Statistical Analysis

Analyses were performed using SPSS Statistics (version 24.0; IBM Corp). Two-sided tests were used throughout, with statistical significance set at *P*<.05.

#### Outcomes

The HAM-D total score was the primary outcome. Secondary outcomes were the HAM-A total score, the DSM-5 Level 2 Depression Scale *T* score, and attendance at scheduled follow-up sessions. Attendance was operationally defined as the participant being present at a scheduled session, whether conducted face-to-face or online, and was recorded at all 7 sessions. Sessions were scheduled at approximately 4-week intervals with a permitted window of ±5 days. When a participant was unable to attend on the scheduled date, the appointment was moved to a date within this window, and the session was conducted; such sessions were recorded as attended. Sessions not conducted within the permitted window were recorded as missed and were not rescheduled outside it. Analyses of the DSM-5 Level 1 domains and all correlation analyses were not prespecified and are reported as exploratory.

#### Sample Characteristics and Baseline Comparisons

Continuous variables were assessed for normality using the Kolmogorov-Smirnov test and are summarized as means and SDs. Baseline between-group comparisons used the independent-samples 2-tailed *t* test or the Mann-Whitney *U* test for continuous variables and the Pearson chi-square test or the Fisher exact test for categorical variables. When expected cell counts were insufficient in contingency tables with more than 2 categories, the Fisher-Freeman-Halton exact test was used.

#### Within-Group Change

Change in symptom severity from baseline to the final assessment was evaluated within each group using the paired-samples 2-tailed *t* test, restricted to participants with data at both time points. Effect sizes are reported as Cohen *d* for paired samples with 95% CIs.

#### Adjusted Between-Group Comparisons

Because allocation was based on patient preference and the groups differed at baseline on the primary outcome, between-group comparisons at each follow-up session were conducted using analysis of covariance adjusting for the corresponding baseline score. A fully adjusted model additionally included baseline HAM-A score, age, sex, education level, and previous psychiatric history. Adjusted mean differences are reported with 95% CIs and partial eta square.

#### Longitudinal Analysis

To use all available repeated observations, linear mixed models were fitted with participant as a random effect and an unstructured covariance matrix. Fixed effects were group, session, and the group-by-time interaction, adjusted for the covariates listed earlier. The group-by-time interaction was used to test whether symptom trajectories differed between the 2 groups over the course of follow-up, and the main effect of time was used to characterize change within groups.

#### Clinical Significance

Response was defined as a reduction of at least 50% in the HAM-D total score from baseline, and remission as a HAM-D total score of 7 or below at the final assessment, in accordance with the severity bands described earlier. Proportions were compared between groups using the Fisher exact test.

#### Missing Data

Missing data arose because participants did not attend scheduled follow-up sessions, in which case, the assessments for that session could not be administered. Reasons for nonattendance were not systematically recorded. The proportion of missing observations is reported for each time point, and the Little test was used to evaluate whether values were missing completely at random. Linear mixed models use all available observations under a missing-at-random assumption. As a sensitivity analysis, multiple imputation was performed by fully conditional specification with 20 imputed datasets, including group, baseline scores, age, sex, education level, and previous psychiatric history in the imputation model, and the adjusted analyses were repeated on the imputed datasets with results pooled using Rubin rules.

Participants who discontinued follow-up were compared with those who completed the study on baseline HAM-D and HAM-A scores, age, sex, previous psychiatric diagnosis, and previous psychiatric treatment, using independent-samples 2-tailed *t* tests and Fisher exact tests as appropriate.

#### Multiplicity

No formal correction for multiple comparisons was applied. Inferential conclusions are restricted to the primary outcome and the prespecified secondary outcomes. Given the number of comparisons involved in the DSM-5 Level 1 domain analyses and the correlation analyses, these results are interpreted descriptively, and no inferential weight is placed on isolated significant findings.

## Results

### Participant Characteristics

Baseline HAM-D scores were higher in the face-to-face group (mean 26.8, SD 3.6) than in the online group (mean 24.4, SD 4.0; t_118_=3.43; *P*=.001), as were baseline DSM-5 Level 2 Depression Scale *T* scores (*P*=.048). Baseline HAM-A scores did not differ between groups (Tables 1-4). Because the groups differed on the primary outcome at baseline, all subsequent between-group comparisons are reported both unadjusted and adjusted for the corresponding baseline value.

**Table 1:** Baseline sociodemographic and clinical characteristics of participants in the face-to-face and online follow-up groups.

Variable	Face-to-face (n=60)	Online (n=60)	Test statistic^a^ (df)	*P* value
Age (mean (SD), range)	36.9 (12.2), 21-64	35.1 (8.7), 21-52	0.96 (118)	.34
Sex, n (%)			–	–
Female	48 (80.0)	48 (80.0)		
Male	12 (20.0)	12 (20.0)		
Education duration, mean (SD), years	9.9 (3.4)	9.8 (3.4)	0.13 (118)	.89
Education level, n (%)			1.3 (3)	.73
Primary	10 (16.7)	8 (13.3)		
Middle	12 (20.0)	15 (25.0)		
High	21 (35.0)	24 (40.0)		
University	17 (28.3)	13 (21.7)		
Marital status, n (%)			0.1 (1)	.71
Married	35 (58.3)	37 (61.7)		
Single	25 (41.7)	23 (38.3)		
Having children, n (%)			0.3 (1)	.58
Yes	37 (61.7)	34 (56.7)		
No	23 (38.3)	26 (43.3)		
Employment status, n (%)			2.3 (1)	.13
Employed	17 (28.3)	25 (41.7)		
Unemployed	43 (71.7)	35 (58.3)		
Monthly income status, n (%)			3.0 (2)	.23
Low income (US $0-$300)	25 (41.7)	19 (31.7)		
Middle income (US $301-602)	18 (30.0)	15 (25.0)		
High income (>US $602)	17 (28.3)	26 (43.3)		
Previous psychiatric diagnosis, n (%)			1.4 (1)	.24
Yes	16 (26.7)	22 (36.7)		
No	44 (73.3)	38 (63.3)		
History of psychiatric treatment, n (%)			1.8 (1)	.18
Yes	5 (8.3)	10 (16.7)		
No	55 (91.7)	50 (83.3)		
Family history of mental illness, n (%)			1.0 (1)	.32
Yes	20 (33.3)	15 (25.0)		
No	40 (66.7)	45 (75.0)		
Smoking, n (%)			2.8 (1)	.10
Yes	20 (33.3)	29 (48.3)		
No	40 (66.7)	31 (51.7)		
Alcohol use, n (%)			1.8 (1)	.18
Yes	10 (16.7)	16 (26.7)		
No	50 (83.3)	44 (73.3)		

^a^Independent-samples 2-tailed t test statistics for continuous variables (degrees of freedom in parentheses) and chi-square statistics for categorical variables. Test statistics and *P* values are shown on the row of each variable and apply to the categories listed beneath it. Dashes indicate not applicable.

### Attrition and Missing Data

In total, 12 of 120 (10%) participants discontinued follow-up entirely, 9 of 60 (15%) in the face-to-face group and 3 of 60 (5%) in the online group. Participants who discontinued did not differ significantly from completers in baseline depression severity (HAM-D: mean 26.7, SD 5.3 vs mean 25.5, SD 3.8; *P*=.46), baseline anxiety severity (HAM-A mean 29.2, SD 5.2 vs mean 26.9, SD 10.5; *P*=.22), age (mean 34.3, SD 11.1 vs mean 36.2, SD 10.6 years; *P*=.58), sex (7/12, 58.3% vs 89/108, 82.4% female; *P*=.06), previous psychiatric diagnosis (3/12, 25% vs 35/108, 32.4%; *P*=.75), or previous psychiatric treatment (2/12, 16.7% vs 13/108, 12%; *P*=.65). Given the small number of participants who discontinued, these comparisons had limited power to detect modest differences.

The proportion of missing HAM-D observations increased over follow-up and was consistently higher in the face-to-face group: session 1: 15% (9/60) versus 5% (3/60), session 2: 23.3% (14/60) versus 8.3% (5/60), session 4: 28.3% (17/60) versus 13.3% (8/60); and session 6: 28.3% (17/60) versus 18.3% (11/60). The Little test indicated that values were not missing completely at random (*χ*^2^_43_=60.4; *P*=.04). Linear mixed models were therefore used as the primary longitudinal analysis, and multiple imputation was performed as a sensitivity analysis.

### Primary Outcome: Depressive Symptoms (HAM-D)

#### Within-Group Change

Depressive symptoms decreased substantially in both groups between baseline and the final assessment. In the face-to-face group, mean HAM-D fell from 26.6 (SD 3.3) to 5.0 (SD 3.7), a reduction of 21.6 points (81.2%; *P*<.001; Cohen *d*=4.86, 95% CI 3.79-5.93; n=43). In the online group, mean HAM-D fell from 24.1 (SD 3.6) to 3.3 (SD 3.2), a reduction of 20.8 points (86.3%; *P*<.001; Cohen *d*=6.06, 95% CI 4.83-7.29; n=49).

#### Between-Group Comparisons

In unadjusted analyses, mean HAM-D scores were lower in the online group at session 4 (mean 6.1, SD 3.7 vs mean 4.6, SD 3.2; *P*=.045) and session 6 (mean 5.0, SD 3.7 vs mean 3.3, SD 3.2; *P*=.02), with no significant differences at sessions 1 or 2 (Table 2). After adjustment for the corresponding baseline HAM-D score, none of these differences remained statistically significant: session 1: +1.25 (95% CI –0.66 to 3.17; *P*=.20), session 2: +0.31 (95% CI –1.67 to 2.29; *P*=.76), session 4: –0.47 (95% CI –1.71 to 0.77; *P*=.46), and session 6: –0.79 (95% CI –2.22 to 0.65; *P*=.28). Partial eta square values were 0.015, 0.001, 0.006, and 0.013, respectively, indicating negligible between-group variance at every time point.

**Table 2:** Hamilton Depression Rating Scale (HAM-D): unadjusted and baseline-adjusted between-group comparisons^a^.

Session	Face-to-face		Online		Unadjusted test statistic	Unadjusted *P* value	Adjusted mean difference (95% CI)^b^	Adjusted *P* value	Partial η^2^
	Values, n	Mean (SD)	Values, n	Mean (SD)					
Zeroth (baseline)	60	26.8 (3.6)	60	24.4 (4.0)	t_118_=3.43^c^	.001	—^d^	—	—
First	51	12.3 (5.9)	57	12.1 (5.6)	t_106_=0.44^c^	.66	+1.25 (–0.66 to 3.17)	.20	0.015
Second	46	9.6 (4.9)	55	8.4 (5.5)	t_99_=1.12^c^	.26	+0.31 (–1.67 to 2.29)	.76	0.001
Fourth	43	6.1 (3.7)	52	4.6 (3.2)	*U*=873^e^	.045	–0.47 (–1.71 to 0.77)	.46	0.006
Sixth	43	5.0 (3.7)	49	3.3 (3.2)	*U*=750.5^e^	.02	–0.79 (–2.22 to 0.65)	.28	0.013

^a^The unadjusted differences observed at sessions 4 and 6 did not persist after adjustment for baseline severity.

^b^Analysis of covariance adjusted for the corresponding baseline HAM-D score. Negative values indicate lower scores in the online group. Fully adjusted results are shown in Table 3.

^c^Independent-samples 2-tailed *t* test statistics for normally distributed data.

^d^Not applicable.

^e^Mann-Whitney *U* statistics for nonnormally distributed data.

In the fully adjusted model, which additionally included baseline HAM-A score, age, sex, years of education, and previous psychiatric history, none of the between-group differences reached significance at any time point: session 1: +1.46 (95% CI –0.55 to 3.46; *P*=.15), session 2: +0.50 (95% CI –1.53 to 2.52; *P*=.63), session 4: –0.61 (95% CI –1.87 to 0.66; *P*=.34), and session 6: –0.40 (95% CI –1.86 to 1.07; *P*=.59). Partial eta square values ranged from 0.003 to 0.021 (Table 3).

**Table 3:** Fully adjusted between-group comparisons for Hamilton Depression Rating Scale (HAM-D) and Hamilton Anxiety Rating Scale (HAM-A)^a^.

Outcome and session		Values, n	Adjusted mean difference^b^	SE^c^	95% CI	*P* value	Partial η^2^
HAM-D							
	First	105	+1.46	1.01	–0.55 to 3.46	.15	0.021
	Second	97	+0.50	1.02	–1.53 to 2.52	.63	0.003
	Fourth	92	–0.61	0.63	–1.87 to 0.66	.34	0.011
	Sixth	88	–0.40	0.74	–1.86 to 1.07	.59	0.004
HAM-A							
	First	104	+0.66	1.36	–2.04 to 3.37	.63	0.002
	Second	98	+0.10	1.36	–2.60 to 2.81	.94	<0.000
	Fourth	92	+0.24	1.41	–2.56 to 3.03	.87	<0.000
	Sixth	89	–0.46	1.50	–3.45 to 2.52	.76	0.001

^a^Model: outcome~group+baseline HAM-D+baseline HAM-A+age+sex+years of education+previous psychiatric history.

^b^Negative values indicate lower scores in the online group. Sex coded 1=female, 2=male; previous psychiatric history coded 1=yes, 2=no; education entered as completed years.

^c^SE of the adjusted mean difference estimated by the analysis of covariance model.

#### Longitudinal Analysis

In the linear mixed model using all available observations, the main effect of time was highly significant, with mean HAM-D decreasing by 2.9 points at session 2, 6.2 points at session 4, and 7.4 points at session 6 relative to session 1 (all *P*<.001). The group-by-time interaction was not significant (*χ*^2^_3_=1.9; *P*=.59), indicating that symptom trajectories did not differ between the 2 modalities.

#### Sensitivity Analysis

Under multiple imputation (*m*=20), the adjusted between-group differences were consistent with the complete-case results: session 4: –0.89 (95% CI –2.10 to 0.32; *P*=.15) and session 6: –0.72 (95% CI –2.03 to 0.59; *P*=.28). The findings were therefore robust to the approach taken to missing data.

#### Severity Categories

At baseline, most participants in the face-to-face group were classified as having moderate depression (43/60, 71.7%), and the remainder as severe (17/60, 28.3%); in the online group, 51 of 60 (85%) were moderate, and 9 of 60 (15%) were severe. Both groups shifted toward lower severity categories over follow-up. At the final session, 32 of 43 (74.4%) of the face-to-face group were in remission, 10 of 43 (23.3%) had mild symptoms, and 1 of 43 (2.3%) remained in the moderate range; in the online group, 43 of 49 (87.8%) were in remission, and the remainder had mild symptoms. Between-group comparison of severity category distributions reached significance only at session 4 (*χ*^2^_1_=4.8; *P*=.03).

#### Clinical Significance

Response, defined as a reduction of at least 50% in HAM-D from baseline, was achieved by 41 of 43 (95.3%) participants in the face-to-face group and 48 of 49 (98%) in the online group (*P*=.60). Remission, defined as a final HAM-D of 7 or below, was achieved by 32 of 43 (74.4%) and 43 of 49 (87.8%), respectively (*P*=.11).

#### Anxiety Symptoms (HAM-A)

Anxiety symptoms also decreased substantially within both groups between baseline and the final assessment: from 27.0 (SD 10.7) to 9.1 (SD 8.0) in the face-to-face group (66.2% reduction; *P*<.001; Cohen *d*=1.61, 95% CI 1.16-2.06) and from 26.4 (SD 10.0) to 7.1(SD 5.1) in the online group (73.4% reduction; *P*<.001; Cohen *d*=2.11, 95% CI 1.60-2.62). No statistically significant between-group differences in HAM-A scores were observed at any time point, either unadjusted (Table 4) or after adjustment for baseline anxiety severity (Table 5): session 1: –1.67 (95% CI –4.45 to 1.12; *P*=.24), session 2: –1.49 (95% CI –4.04 to 1.06; *P*=.25), session 4: –0.71 (95% CI –3.33 to 1.92; *P*=.59), and session 6: –1.97 (95% CI –4.68 to 0.74; *P*=.15). Partial eta square values were 0.014, 0.014, 0.003, and 0.023, respectively (Tables 4 and 5).

**Table 4:** Hamilton Anxiety Rating Scale (HAM-A) results.

Session	Face-to-face		Online		Test statistic^a^	*P* value
	Values, n	Mean (SD)	Values, n	Mean (SD)		
Zeroth (baseline)	60	26.4 (9.4)	60	26.8 (10.0)	1844.0	.82
First	51	16.1 (9.7)	57	15.1 (6.7)	1471.0	.78
Second	46	12.8 (8.4)	55	11.7 (6.0)	1430.0	.26
Fourth	43	9.6 (8.6)	52	9.3 (4.3)	1046.0	.59
Sixth	43	9.1 (8.0)	49	7.1 (5.1)	1146.5	.47

^a^Values in the test statistic column represent Mann-Whitney *U* statistics.

**Table 5:** Hamilton Anxiety Rating Scale (HAM-A): baseline-adjusted between-group comparisons^a^.

Session	Adjusted mean difference (95% CI)^b^	Adjusted *P* value	Partial η^2^
First	−1.67 (−4.45 to 1.12)	.24	0.014
Second	−1.49 (−4.04 to 1.06)	.25	0.014
Fourth	−0.71 (−3.33 to 1.92)	.59	0.003
Sixth	−1.97 (−4.68 to 0.74)	.15	0.023

^a^Analysis of covariance adjusted for the corresponding baseline HAM-A score.

^b^Negative values indicate lower scores in the online group.

In the fully adjusted model, between-group differences in HAM-A scores were negligible at every time point: session 1: +0.66 (95% CI –2.04 to 3.37; *P*=.63), session 2: +0.10 (95% CI –2.60 to 2.81; *P*=.94), session 4: +0.24 (95% CI –2.56 to 3.03; *P*=.87), and session 6: –0.46 (95% CI –3.45 to 2.52; *P*=.76). Partial eta square values were 0.002 or lower at all time points (Table 3).

Between-group comparison of HAM-A severity category distributions reached significance only at session 1 (*χ*^2^_3_=8.6; *P*=.03).

#### Self-Reported Depressive Symptoms (DSM-5 Level 2)

Unadjusted DSM-5 Level 2 *T* scores were lower in the online group at session 4 (*P*=.007) and session 6 (*P*=.003). After adjustment for the corresponding baseline score, the difference remained significant at session 4 (–2.52, 95% CI –4.56 to –0.48; *P*=.02) but not at session 1 (+0.90, 95% CI –1.78 to 3.58; *P*=.51) or session 6 (–1.45, 95% CI –3.34 to 0.45; *P*=.13). In the linear mixed model, the group-by-time interaction was not significant (*P*=.12). This isolated finding is discussed further in the Divergence Between Clinician-Rated and Self-Reported Measures section.

#### Exploratory Analyses: DSM-5 Level 1 Cross-Cutting Symptom Measure

Analyses of the 13 DSM-5 Level 1 domains were not prespecified and involved a large number of comparisons across domains and time points; they are therefore reported descriptively in Multimedia Appendix 3, and no inferential weight is placed on individual findings. Both groups showed a reduction over follow-up in the proportion of participants screening above threshold for depression and anxiety. At baseline, all participants in both groups screened above the depression threshold. A higher proportion of the face-to-face group screened above the mania threshold (9/60, 15% vs 2/60, 3.3%; *P*=.03), and a substantial proportion of both groups screened above the psychosis threshold (14/60, 23.3% vs 17/60, 28.3%; *P*=.53), despite the exclusion of psychotic and bipolar disorders at eligibility assessment. None of these positive screens was confirmed as a psychotic or manic syndrome on clinical evaluation, consistent with the poor positive predictive power of this instrument.

#### Exploratory Correlation Analyses

Correlations between sociodemographic variables and symptom scores were not prespecified and are reported in Multimedia Appendix 4. Female sex and married status were associated with higher HAM-D scores at several time points. At session 6, HAM-A scores showed negative correlations with self-reported alcohol use (ρ=–0.32; *P*=.04) and cigarette use (ρ=–0.39; *P*=.01), indicating lower anxiety scores among participants reporting such use; these associations were not observed at any other time point. Given the number of correlations examined, these findings are treated as hypothesis-generating and are interpreted with caution in the Discussion section.

#### SPS

Baseline SPS subscale scores did not differ significantly between groups on any subscale (Table 6). Scores were low in both groups and well below the levels reported in clinical samples characterized by elevated suicide risk, consistent with the exclusion of patients with active suicidal ideation at eligibility assessment. The hopelessness subscale is reported both with and without items 5 and 19; the between-group comparison was unaffected by this choice.

**Table 6:** Suicide Probability Scale subscale scores in the face-to-face and online groups.

Subscale	Face-to-face, mean (SD)	Online, mean (SD)	Test statistic	*P* value
Hopelessness	30.5 (7.2)	29.5 (6.3)	t_118_=0.79^a^	.43
Hopelessness (excluding items 5 and 19)	24.7 (6.5)	23.6 (5.8)	t_118_=1.03^a^	.31
Hostility	13.4 (3.5)	12.9 (3.9)	*U*=1983.5^b^	.33
Suicidal ideation	11.7 (3.1)	11.4 (3.1)	*U*=1889.5^b^	.64
Negative self-evaluation	19.1 (4.5)	20.0 (4.1)	*U*=1622.5^b^	.35

^a^Independent-samples 2-tailed *t* test statistics for normally distributed data.

^b^Mann-Whitney *U* statistics for nonnormally distributed data.

#### Pharmacological Treatment

All participants received antidepressant pharmacotherapy, initiated in the outpatient clinic. Antidepressant class at baseline was comparable between groups, with 56 and 57 participants, respectively, receiving a selective serotonin reuptake inhibitor (SSRI) and 4 and 3 a serotonin-norepinephrine reuptake inhibitor (*P*>.99), as was the distribution of active agents (sertraline: n=30 vs n=30, escitalopram: n=13 vs n=13, fluoxetine: n=10 vs n=11; *P*=.78). The most frequently prescribed agents at baseline were sertraline 50 mg (30/60, 50% in each group), escitalopram 5-10 mg (13/60, 21.7% in each group), and fluoxetine 20 mg (10/60, 16.7% vs 11/60, 18.3%). Doses were titrated upward over follow-up in both groups; at the final session, the corresponding figures were sertraline 100-200 mg (22/43, 51.2%) and escitalopram 10-20 mg (9/43, 20.9%) in the face-to-face group, and sertraline 75-150 mg (22/49, 44.9%) and escitalopram 10-15 mg (11/49, 22.5%) in the online group.

During follow-up, the 2 groups did not differ in the proportion of participants with at least 1 dose increase (40/60, 66.7% vs 36/60, 60%; *P*=.57), addition of a second agent (9/60, 15% vs 3/60, 5%; *P=.*13), augmentation (7/60, 11.7% vs 4/60, 6.7%; *P*=.53), prescription of a hypnotic (8/60, 13.3% vs 10/60, 16.7%; *P*=.80), or documented nonadherence (5/60, 8.3% vs 9/60, 15%; *P*=.39; Table 7).

**Table 7:** Pharmacological treatment at baseline and during follow-up.

Variable		Face-to-face (n=60), n (%)	Online (n=60), n (%)	*P* value	
Antidepressant class at baseline^a^					>.99
	SSRI^b^	56 (93.3)	57 (95)		
	SNRI^c^	4 (6.7)	3 (5)		
Active agent at baseline					.78
	Sertraline	30 (50)	30 (50)		
	Escitalopram	13 (21.7)	13 (21.7)		
	Fluoxetine	10 (16.7)	11 (18.3)		
During follow-up					
	At least 1 dose increase	40 (66.7)	36 (60)	.57	
	Addition of a second agent	9 (15)	3 (5)	.13	
	Augmentation	7 (11.7)	4 (6.7)	.53	
	Hypnotic prescribed	8 (13.3)	10 (16.7)	.80	
	Documented nonadherence	5 (8.3)	9 (15)	.39	

^a^Mirtazapine at doses of 30 mg or above was classified as an antidepressant and doses of 15 mg or below as a hypnotic; trazodone was classified as a hypnotic in all cases.

^b^SSRI: selective serotonin reuptake inhibitor.

^c^SNRI: serotonin-norepinephrine reuptake inhibitor.

#### Attendance

Attendance across all 7 scheduled sessions was higher in the online group overall: 373 of 420 (88.8%) scheduled sessions were attended, compared with 319 of 420 (76%) in the face-to-face group (*χ*^2^_1_=23.9; *P*<.001). Session-by-session differences reached statistical significance at sessions 2 through 5, with odds ratios ranging from 2.35 to 4.17 (95% CIs are given in Table 8). Differences at session 1 (*P*=.07) and session 6 (*P*=.20) did not reach significance. Because reasons for nonattendance were not recorded, it is not possible to determine whether missed sessions reflected symptomatic improvement, logistical barriers, or other factors.

**Table 8:** Attendance at scheduled follow-up sessions.

Session	Face-to-face, n (%)	Online, n (%)	Chi-square^a^ (*df*=1)	*P* value	OR^b,c^ (95% CI)
Zeroth (baseline)	60 (100)	60 (100)	—^d^	—	—
First	51 (85)	57 (95)	3.3	.07	3.35 (0.86-13.07)
Second	46 (76.7)	55 (91.7)	5.1	.02	3.35 (1.12-9.99)
Third	41 (68.3)	54 (90)	8.5	.003	4.17 (1.53-11.38)
Fourth	43 (71.7)	52 (86.7)	4.1	.04	2.57 (1.01-6.53)
Fifth	35 (58.3)	46 (76.7)	4.6	.03	2.35 (1.07-5.16)
Sixth	43 (71.7)	49 (81.7)	1.7	.20	1.76 (0.74-4.17)
Total	319/420 (76)	373/420 (88.8)	23.9	<.001	—

^a^Pearson chi-square test; all expected cell counts >5.

^b^OR: odds ratio.

^c^Odds of attending in the online group relative to the face-to-face group.

^d^Not applicable.

## Discussion

### Principal Findings

In this prospective observational cohort of 120 outpatients with major depressive disorder followed for 6 months, depressive symptoms decreased substantially in both the face-to-face and the online follow-up groups, with reductions exceeding 80% of baseline severity and large within-group effect sizes. In unadjusted comparisons, HAM-D scores were lower in the online group at the fourth and sixth sessions. However, the groups differed on the primary outcome at baseline, and after adjustment for baseline severity, none of these differences remained statistically significant; the corresponding partial eta square values were below 0.03 at every time point. A linear mixed model using all available observations showed a strong effect of time but no group-by-time interaction, indicating that symptom trajectories did not differ between modalities. Rates of response and remission were high in both groups and did not differ significantly. The most consistent difference between the groups was attendance: participants in the online group attended a higher proportion of scheduled sessions overall.

These findings do not establish that the 2 modalities are equivalent. Demonstrating equivalence requires a predefined equivalence margin and CI-based testing, as applied by O’Reilly et al [14] in a randomized equivalence trial; the absence of a statistically significant difference in an observational study of this size cannot be interpreted as evidence of equivalence. The study is therefore best understood as a descriptive account of the feasibility of online psychiatric follow-up and of its association with continuity of care, rather than as a comparative effectiveness claim.

To our knowledge, this is one of the few longitudinal comparisons of face-to-face and online psychiatric follow-up among adults with major depressive disorder conducted in Türkiye.

### Comparison With Prior Studies

The substantial symptom reduction observed in both groups is consistent with the wider literature on telepsychiatry for depression. In a meta-analysis of randomized controlled trials, Hagi et al [10] found no significant difference between telepsychiatry and face-to-face treatment when all diagnoses were pooled (k=26; n=2290; *P*=.25). A superiority of telepsychiatry was reported for depressive disorders specifically (standardized mean difference –0.325, 95% CI –0.640 to –0.011; *P*=.04), but this estimate rested on 6 trials, and its CI only marginally excluded 0. The same analysis found that the effect size for depressive disorders increased with longer trial duration, with no significant difference in trials of 26 weeks or shorter, and superiority emerging only in trials of 36 weeks or longer [10]. The 6-month duration of the present study falls within the range in which comparable outcomes would be expected, and our findings are consistent with that pattern.

A meta-analysis of randomized trials restricted to patients with depression similarly reported that real-time telehealth may be a viable alternative to face-to-face care, with no difference in treatment satisfaction (standardized mean difference –0.14, 95% CI –0.56 to 0.28; *P*=.51) [13]. In the randomized equivalence trial by O’Reilly et al [14], psychiatric consultation and short-term follow-up delivered by telepsychiatry produced clinical outcomes equivalent to those achieved face-to-face, with similar levels of patient satisfaction. A review by Hilty et al [32] concluded that telemental health is effective across populations and settings, appears comparable to in-person care, and improves access to services, particularly for rural populations underserved by psychiatry. Beyond symptom outcomes, videoconferencing-based psychiatric assessments have been reported to be reliable, and telepsychiatric care has been associated with high levels of patient satisfaction and with clinical outcomes comparable to conventional treatment across diverse patient populations [33]. The feasibility of rapid implementation has also been demonstrated in routine settings: one North American outpatient psychiatric clinic converted from almost entirely in-person to fully virtual delivery within 3 business days at the onset of the COVID-19 pandemic [34]. Our finding of no significant difference in anxiety symptoms is consistent with the meta-analysis by Shaker et al [35], which found no difference in symptom reduction between telemedicine and in-person treatment across mood and anxiety disorders.

### Divergence Between Clinician-Rated and Self-Reported Measures

The 3 depression measures used in this study did not converge. On the clinician-rated HAM-D, the between-group difference narrowed relative to baseline and did not survive adjustment. On the self-reported DSM-5 Level 2 Depression Scale, by contrast, the difference at the fourth session persisted after adjustment for baseline severity (–2.52, 95% CI –4.56 to –0.48; *P*=.02), although the group-by-time interaction in the mixed model was not significant.

Several explanations are possible. First, the 2 instruments differ in perspective rather than in reference period: both cover the preceding week, but the HAM-D is rated by the clinician on the basis of the clinical interview, whereas the Level 2 measure is completed by the patient; discrepancies between clinician-rated and self-reported depression severity are well documented and tend to be larger in the mid-treatment phase. Second, the assessing clinician could not be blinded to follow-up modality, which introduces a potential source of measurement bias in the clinician-rated instruments that does not apply to the self-report measures. Third, the setting in which self-report forms were completed differed between groups: face-to-face participants completed printed forms in the clinic, whereas online participants completed them electronically at home, and this difference in administration context may itself have influenced responding. Finally, given the number of comparisons undertaken, an isolated significant result at a single time point may reflect chance. Because the HAM-D was the prespecified primary outcome and the Level 2 finding was neither replicated at the sixth session nor supported by the longitudinal model, we do not interpret it as evidence of a modality effect. This divergence nevertheless illustrates the limitation of relying on a single instrument to characterize symptom change and supports the use of a prespecified primary outcome.

The DSM-5 Level 1 measure is a screening instrument rather than a severity index. Dichotomizing its domains at screening thresholds and comparing proportions across time provides only a coarse indication of symptom burden, and the associated analyses were exploratory. The elevated proportion of participants screening above the mania threshold at baseline, despite the exclusion of psychotic and bipolar disorders, is consistent with reports that this instrument has poor sensitivity and positive predictive power across symptom domains because of a high rate of false-positive responses [36]. In every such case in the present study, the score corresponded to the lowest severity level of the item and was not confirmed on clinical assessment.

### Exploratory Findings

Several exploratory observations warrant brief comment, with the caveat that they arise from a large number of unplanned comparisons and were not corrected for multiplicity.

At baseline, a significantly higher proportion of the face-to-face group screened above the mania threshold of the DSM-5 Level 1 measure. Although no participant met criteria for bipolar disorder and none developed a manic syndrome during follow-up, subthreshold manic or hypomanic features have been associated with a less favorable illness course and with differences in antidepressant response, and this baseline imbalance may therefore have contributed to the slower symptomatic improvement observed in that group. The mania domain was not included as a covariate in the adjusted models, and this possibility cannot be excluded.

At the final session, HAM-A scores were negatively correlated with self-reported alcohol and cigarette use, indicating lower anxiety scores among participants reporting such use. This association was confined to a single time point among 5 and is most plausibly a chance finding. Should it reflect a real pattern, several explanations are possible: substances may be used to relieve anxiety, with lower residual symptoms among those continuing to use them; participants with lower anxiety may be less avoidant and therefore more likely to engage in social behaviors involving alcohol or tobacco; or reporting of both substance use and anxiety may be influenced by common response tendencies. Use was recorded as a binary variable without quantity or frequency, and the study was not designed to examine this relationship; the finding should be regarded as hypothesis-generating rather than as evidence of an association between substance use and anxiety.

The remaining correlations, including those between HAM-D scores and sex, marital status, and number of children, are consistent with well-documented sociodemographic patterns in depression and are reported for completeness rather than interpreted substantively.

### Attendance and Continuity of Care

Attendance was the most consistent difference between the 2 groups, with 88.8% (373/420) of scheduled sessions attended in the online group compared with 76% (319/420) in the face-to-face group. Session-by-session differences reached significance at sessions 2 through 5.

This finding should be interpreted with care. Attendance records whether a participant was present at a scheduled session; it does not measure engagement with treatment, and higher attendance does not by itself indicate a greater therapeutic effect. Practical factors are plausible contributors: online sessions require no travel, involve less disruption to work and daily routine, and can be attended from home. Because reasons for nonattendance were not recorded in this study, we cannot determine whether missed sessions reflected symptomatic improvement, logistical barriers, dissatisfaction, or other factors, and the explanations above remain hypotheses rather than findings.

Comparable observations have been reported elsewhere. In a cohort of 3476 patients at 3 outpatient psychiatric clinics, the transition from in-person care to teletherapy was accompanied by a reduction in missed appointments and in depressive symptom scores, although the pre- and postdesign of that study does not permit a between-modality comparison [37]. In a rural community-based rehabilitation program in Karnataka, India, a shift to video consultations during pandemic lockdowns maintained continuity of care for more than 80% of persons with serious mental illness at a time when travel restrictions prevented attendance at in-person clinics [6]. Engagement with remotely delivered care under routine conditions is generally lower than in trial settings: in a meta-analysis of guided internet-based cognitive behavioral therapy delivered in routine care, the average proportion of sessions completed was 62.6% in depression studies and 57.3% in anxiety studies, compared with approximately 80% reported in randomized trials, a difference the authors attributed to the adherence-fostering effect of controlled research settings [15]. Although session completion in self-guided internet-based therapy is not directly equivalent to attendance at clinician-led consultations, the attendance observed in the online group of the present study compares favorably with these routine-care benchmarks.

### Context in Türkiye

Mental health services in Türkiye remain predominantly hospital-based, and community-oriented and digital services are still limited [38]. Stigma and the limited development of community-based services have been identified as barriers to equitable care [39], and the accessibility and privacy afforded by remote consultation have been discussed as potentially relevant to help-seeking in this context [8]. These considerations were not measured in the present study and are offered as contextual background rather than as explanations of our findings.

### Strengths

This study has several strengths. It is one of the first longitudinal comparisons of online and face-to-face psychiatric follow-up among adults with major depressive disorder in Türkiye. It was conducted under routine outpatient conditions rather than as a structured psychotherapy trial, which increases the ecological validity of the findings. All clinician-rated assessments were performed by a single psychiatrist in both groups, so that treatment modality was not confounded with rater. The assessment battery combined clinician-rated and self-report instruments, allowing the convergence of these perspectives to be examined. Concomitant pharmacological treatment was documented and compared between groups. Finally, operational procedures for online psychiatric consultation, including information delivery, privacy safeguards, and a predefined protocol for emergent suicide risk, were established and are reported in full.

### Limitations

Several limitations should be considered. First, allocation was based on patient preference rather than randomization. Patients who choose remote follow-up may differ systematically from those who prefer in-person visits in technological familiarity, socioeconomic circumstances, treatment expectations, or perceived accessibility, and such differences may not be captured by the variables measured here. Baseline differences on the primary outcome were present and were addressed by adjustment, but residual confounding cannot be excluded, and the findings represent associations rather than causal effects of delivery modality. Three steps were taken to limit the influence of preference-based allocation. The groups were compared at baseline on all measured sociodemographic and clinical variables and differed only on depressive symptom severity (Table 1); every between-group comparison was adjusted for the corresponding baseline score, with a fully adjusted model additionally including baseline anxiety severity, age, sex, years of education, and previous psychiatric history; and prescribing was carried out by the same pool of clinicians in both groups, so that pharmacological management was not tied to modality. These steps address measured confounding only. Digital literacy, comfort with videoconferencing, and motivation for treatment were not assessed, and any imbalance in these characteristics would remain uncontrolled. A meta-synthesis comparing patients enrolled in eHealth and face-to-face psychotherapy trials for depression found that most baseline characteristics were broadly similar but that patients in eHealth trials reported a longer duration of depression and were less likely to have received previous treatment [17]; comparable differences may have been present in this sample.

Second, the study was not designed as an equivalence trial, and the absence of statistically significant between-group differences cannot be interpreted as evidence of equivalence.

Third, the assessing clinician could not be blinded to follow-up modality, which introduces a potential source of measurement bias in the clinician-rated instruments.

Fourth, attrition was greater in the face-to-face group, and the proportion of missing observations increased over follow-up. The Little test indicated that data were not missing completely at random. Participants who discontinued did not differ significantly from completers on the variables examined, and mixed models and multiple imputation produced consistent results; nevertheless, attrition bias cannot be fully excluded, and the comparison of discontinuers with completers had limited power.

Fifth, reasons for nonattendance were not recorded, so the interpretation of the attendance finding is necessarily limited.

Sixth, a large number of comparisons were undertaken across symptom domains and time points. No formal correction for multiplicity was applied; inferential conclusions are restricted to the primary and prespecified secondary outcomes, and the exploratory analyses should be regarded as hypothesis-generating.

Seventh, diagnoses were established through clinical evaluation according to DSM-5 criteria without a structured diagnostic interview, which may have introduced diagnostic heterogeneity.

Eighth, the sample size was determined on the assumption of a medium effect size (Cohen *d*=0.50), and the face-to-face group fell below the required 51 participants at the second, fourth, and sixth sessions. The between-group differences actually observed were smaller than the value assumed at the design stage, and the study was correspondingly underpowered to detect differences of that magnitude.

Ninth, pharmacological treatment followed routine clinical practice rather than a standardized protocol. Antidepressant class, active agent, and prescribing changes were comparable between groups, and prescribing was carried out by the same pool of clinicians irrespective of modality; nevertheless, medication was not randomized and remains a potential source of confounding, since roughly two-thirds of the total reduction in HAM-D occurred between baseline and the first follow-up session, in the window in which treatment was initiated and first adjusted.

Tenth, no structured psychotherapy protocol was delivered, and session content followed routine practice, so the content of what was compared was not standardized beyond the common interview framework.

Eleventh, participants were recruited at a single tertiary university hospital, the sample was predominantly female, and individuals without access to the equipment required for videoconferencing were ineligible, which limits generalizability and reflects a wider concern about digital inequality.

### Conclusions

Among outpatients with major depressive disorder followed for 6 months under routine conditions, both online and face-to-face psychiatric follow-up were accompanied by large reductions in depressive and anxiety symptoms, and between-group differences in symptom outcomes did not persist after adjustment for baseline severity. Online psychiatric follow-up was associated with clinical outcomes comparable to those observed in face-to-face care in this sample; however, equivalence cannot be established due to the observational design. The most consistent difference favoring online follow-up was attendance at scheduled sessions, although the reasons for this difference could not be determined. These findings characterize online psychiatric follow-up as a feasible means of maintaining continuity of routine care rather than as a more effective one. Randomized trials with predefined equivalence margins, standardized session content, systematic recording of reasons for nonattendance, and longer follow-up are needed to determine whether the 2 modalities are genuinely comparable and to establish the cost-effectiveness of remote psychiatric follow-up.

## Data Availability

The datasets generated and analyzed during this study are not publicly available because they contain clinical information from individual psychiatric outpatients, and the informed consent obtained from participants did not include provision for public data sharing. Deidentified data supporting the findings may be made available from the corresponding author on reasonable request.

## Appendix

Multimedia Appendix 1 STROBE checklist.

Multimedia Appendix 2 Structured psychiatric interview protocol.

Multimedia Appendix 3 Diagnostic and Statistical Manual of Mental Disorders, Fifth Edition Level 1 Cross-Cutting Symptom Measure: domain-level results.

Multimedia Appendix 4 Spearman correlations between symptom scores and sociodemographic variables.

## Abbreviations

DSM-5: Diagnostic and Statistical Manual of Mental Disorders, Fifth Edition
HAM-A: Hamilton Anxiety Rating Scale
HAM-D: Hamilton Depression Rating Scale
SPS: Suicide Probability Scale
STROBE: Strengthening the Reporting of Observational Studies in Epidemiology
