# Supplementary material for: Symptom Trajectories and Attendance in Face-to-Face and Online Psychiatric Follow-Up for Major Depressive Disorder and Anxiety Symptoms: Prospective Observational Cohort Study
Source: JMIR Form Res. 2026 Oct 19;10:e97656. doi: 10.2196/97656 (PMC13728771; doi:10.2196/97656)
Supplement: Supplementary file 1 [file formative_v10i1e97656_app1.docx]

|  | Item No. | Recommendation | Page No. | Relevant text from manuscript |
| --- | --- | --- | --- | --- |
| Title and abstract | 1 | (a) Indicate the study's design with a commonly used term in the title or the abstract | 1 | Abstract, Methods: "In this prospective, non-randomized observational cohort study, 120 adults with DSM-5 major depressive disorder were followed for six months at a university hospital psychiatry department in Ankara, Türkiye (September 2021 to May 2022)." |
|  | 1 | (b) Provide in the abstract an informative and balanced summary of what was done and what was found | 1 | Abstract, Results: "Depressive symptoms decreased substantially in both groups: mean HAM-D fell from 26.6 to 5.0 in the face-to-face group (81.2% reduction; Cohen d=4.86; P<.001; n=43) and from 24.1 to 3.3 in the online group (86.3% reduction; Cohen d=6.06; P<.001; n=49)... Unadjusted between-group differences at sessions 4 and 6 did not persist after adjustment for baseline severity (session 4: -0.47, 95% CI -1.71 to 0.77, P=.46; session 6: -0.79, 95% CI -2.22 to 0.65, P=.28), and the group-by-time interaction was not significant (χ²=1.91, df=3, P=.59)... Attendance was higher in the online group overall (373/420, 88.8% vs 319/420, 76.0%; P<.001) and at sessions 2 through 5." |
| **Introduction** |  |  |  |  |
| Background/rationale | 2 | Explain the scientific background and rationale for the investigation being reported | 2-3 | "Mental health professionals play a critical role in the diagnosis, treatment, and follow-up of these conditions; however, access to specialist care remains constrained in many settings... A further limitation is that most of this evidence derives from randomized trials, in which allocation is controlled and the structured research environment may itself foster adherence; how the two modalities compare under routine outpatient conditions, where patients choose their own follow-up modality, has been less well described." |
| Objectives | 3 | State specific objectives, including any prespecified hypotheses | 3-4 | "The aim of this study was to describe symptom trajectories, treatment continuity, and attendance over six months of routine psychiatric follow-up delivered either face to face or online to adults with major depressive disorder, and to examine whether outcomes differed between the two modalities after adjustment for baseline symptom severity and other potential confounders. Because allocation was determined by patient preference rather than randomization, the study was designed as an observational comparison; it was not intended to establish equivalence between modalities, and the findings are interpreted as associations rather than as causal effects of delivery modality." No hypothesis was prespecified. |
| **Methods** |  |  |  |  |
| Study design | 4 | Present key elements of study design early in the paper | 3 | "This was a prospective, non-randomized observational cohort study conducted at the Department of Psychiatry, Ankara University Faculty of Medicine, a tertiary university hospital in Ankara, Türkiye... Because allocation was based on patient preference rather than randomization, the study was designed and is reported as an observational comparison; findings are interpreted as associations rather than as evidence of a causal effect of delivery modality." |
| Setting | 5 | Describe the setting, locations, and relevant dates, including periods of recruitment, exposure, follow-up, and data collection | 3-4 | "Participants were recruited between September 2021 and May 2022 from patients presenting to the adult psychiatry outpatient clinic." "After the baseline interview (session 0), six follow-up sessions were scheduled at approximately 4-week intervals, giving a total of seven sessions over six months per participant." Face-to-face sessions were conducted in the outpatient clinic; online sessions by Zoom or WhatsApp video call. |
| Participants | 6 | (a) Cohort study—Give the eligibility criteria, and the sources and methods of selection of participants. Describe methods of follow-up | 4 | "Of 125 patients assessed for eligibility, 5 were excluded because of active suicidal ideation. The remaining 120 provided written informed consent and were enrolled. Each participant selected the follow-up modality themselves, resulting in 60 participants in the face-to-face group and 60 in the online group." Inclusion and exclusion criteria are listed in full in the same subsection. "The intention was to invite all consecutive patients meeting the eligibility criteria... recruitment is therefore best described as consecutive in intention but incomplete in practice." |
|  | 6 | (b) Cohort study—For matched studies, give matching criteria and number of exposed and unexposed | - | Not applicable (participants were not matched). |
| Variables | 7 | Clearly define all outcomes, exposures, predictors, potential confounders, and effect modifiers. Give diagnostic criteria, if applicable | 7-9 | "The HAM-D total score was the primary outcome. Secondary outcomes were the HAM-A total score, the DSM-5 Level 2 Depression Scale T score, and attendance at scheduled follow-up sessions." Covariates: baseline HAM-D, baseline HAM-A, age, sex, years of education, previous psychiatric history. Diagnoses were established according to DSM-5 criteria (Diagnostic Assessment). |
| Data sources/ measurement | 8* | For each variable of interest, give sources of data and details of methods of assessment (measurement). Describe comparability of assessment methods if there is more than one group | 7-8 | All instruments are described in the Measures subsection. "All study interviews and clinician-rated assessments (HAM-D and HAM-A) were conducted by a single psychiatrist (the first author) in both groups throughout the six-month follow-up period." "The timing, structure, and clinical content of sessions were identical in the two groups, and the mode of delivery was the only intended difference between them." |
| Bias | 9 | Describe any efforts to address potential sources of bias | 3, 8-9 | Selection bias is addressed by adjustment: "Because allocation was based on patient preference and the groups differed at baseline on the primary outcome, between-group comparisons at each follow-up session were conducted using analysis of covariance adjusting for the corresponding baseline score. A fully adjusted model additionally included baseline HAM-A score, age, sex, education level, and previous psychiatric history." Rater bias is limited by the use of a single rater across both groups; the absence of blinding is reported and discussed in the Limitations. |
| Study size | 10 | Explain how the study size was arrived at | 10 | The sample size was determined a priori to detect a medium effect size (Cohen d=0.50) with 80% power at alpha=.05, requiring 51 participants per group. The Limitations note that the face-to-face group fell below this threshold at sessions 4 and 6. |
| Quantitative variables | 11 | Explain how quantitative variables were handled in the analyses. If applicable, describe which groupings were chosen and why | 7-9 | HAM-D and HAM-A total scores and DSM-5 Level 2 T scores were analysed as continuous variables. HAM-D severity bands were defined by the empirically derived cutoffs of Zimmerman et al: no depression (0-7), mild (8-16), moderate (17-23), severe (>=24). DSM-5 Level 1 domains were dichotomised at the recommended screening thresholds and are reported descriptively only. |
| Statistical methods | 12 | (a) Describe all statistical methods, including those used to control for confounding | 9-10 | "Analyses were performed using IBM SPSS Statistics version 24.0 (IBM Corp). Two-sided tests were used throughout, with statistical significance set at P<.05." Baseline comparisons used t tests, Mann-Whitney U tests, chi-square and Fisher exact tests. Within-group change was assessed by paired-samples t tests with Cohen d and 95% CIs. Between-group comparisons used analysis of covariance adjusted for baseline score, with a fully adjusted model including the covariates listed above. |
|  | 12 | (b) Describe any methods used to examine subgroups and interactions | 10 | "To use all available repeated observations, linear mixed models were fitted with participant as a random effect and an unstructured covariance matrix. Fixed effects were group, session, and the group-by-time interaction, adjusted for the covariates listed above... The group-by-time interaction was used to test whether symptom trajectories differed between the two groups over the course of follow-up, and the main effect of time was used to characterize change within groups." No subgroup analyses were performed. |
|  | 12 | (c) Explain how missing data were addressed | 10 | "Missing data arose because participants did not attend scheduled follow-up sessions... The proportion of missing observations is reported for each time point, and Little's test was used to evaluate whether values were missing completely at random. Linear mixed models use all available observations under a missing-at-random assumption. As a sensitivity analysis, multiple imputation was performed by fully conditional specification with 20 imputed datasets... with results pooled using Rubin rules." |
|  | 12 | (d) Cohort study—If applicable, explain how loss to follow-up was addressed | 10 | "Participants who discontinued follow-up were compared with those who completed the study on baseline HAM-D and HAM-A scores, age, sex, previous psychiatric diagnosis, and previous psychiatric treatment, using independent-samples t tests and Fisher exact tests as appropriate." |
|  | 12 | (e) Describe any sensitivity analyses | 10 | Multiple imputation (m=20) was performed as a sensitivity analysis for the adjusted between-group comparisons. |
| **Results** |  |  |  |  |
| Participants | 13* | (a) Report numbers of individuals at each stage of study | 4, 12 | "Of 125 patients assessed for eligibility, 5 were excluded because of active suicidal ideation. The remaining 120 provided written informed consent and were enrolled... 60 participants in the face-to-face group and 60 in the online group.... Twelve participants (10.0%) discontinued follow-up entirely, 9 (15.0%) in the face-to-face group and 3 (5.0%) in the online group." Attendance at each session is reported in Table 8. |
|  | 13* | (b) Give reasons for non-participation at each stage | 4, 12 | Five patients were excluded for active suicidal ideation. "Patients who declined were treated according to routine clinical practice; the number of patients who declined and their reasons were not systematically recorded." "Reasons for non-attendance were not systematically recorded." |
|  | 13* | (c) Consider use of a flow diagram | 5 | Figure 1 (participant flow diagram, Methods). |
| Descriptive data | 14* | (a) Give characteristics of study participants and information on exposures and potential confounders | 11, Table 1 | Table 1 reports age, sex, education, marital status, children, employment, income, previous psychiatric diagnosis and treatment, family history, smoking and alcohol use for both groups. "Baseline HAM-D scores were higher in the face-to-face group (26.8, SD 3.6) than in the online group (24.4, SD 4.0; t₁₁₈=3.43, P=.001), as were baseline DSM-5 Level 2 Depression Scale T scores (P=.048)." |
|  | 14* | (b) Indicate number of participants with missing data for each variable of interest | 12 | "The proportion of missing HAM-D observations increased over follow-up and was consistently higher in the face-to-face group: 15.0% versus 5.0% at session 1, 23.3% versus 8.3% at session 2, 28.3% versus 13.3% at session 4, and 28.3% versus 18.3% at session 6." |
|  | 14* | (c) Cohort study—Summarise follow-up time | 4, 6 | All participants were followed for six months, with seven scheduled sessions at approximately 4-week intervals (window +/-5 days). |
| Outcome data | 15* | Cohort study—Report numbers of outcome events or summary measures over time | 13-18, Tables 2-8 | HAM-D, HAM-A and DSM-5 Level 2 scores at each assessment are reported in Tables 2, 4 and in the text. "Response, defined as a reduction of at least 50% in HAM-D from baseline, was achieved by 41/43 participants (95.3%) in the face-to-face group and 48/49 (98.0%) in the online group (P=.60). Remission, defined as a final HAM-D of 7 or below, was achieved by 32/43 (74.4%) and 43/49 (87.8%) respectively (P=.11)." |
|  | 15* | Case-control study—Report numbers in each exposure category | - | Not applicable. |
|  | 15* | Cross-sectional study—Report numbers of outcome events or summary measures | - | Not applicable. |
| Main results | 16 | (a) Give unadjusted estimates and, if applicable, confounder-adjusted estimates and their precision. Make clear which confounders were adjusted for and why they were included | 13-15, Tables 2-3 | Table 2 reports unadjusted and baseline-adjusted comparisons side by side; Table 3 reports fully adjusted models. "After adjustment for the corresponding baseline HAM-D score, none of these differences remained statistically significant: session 1, +1.25 (95% CI -0.66 to 3.17; P=.20); session 2, +0.31 (95% CI -1.67 to 2.29; P=.76); session 4, -0.47 (95% CI -1.71 to 0.77; P=.46); session 6, -0.79 (95% CI -2.22 to 0.65; P=.28)." Covariates were selected a priori as the variables on which the groups differed at baseline or which are established prognostic factors in depression. |
|  | 16 | (b) Report category boundaries when continuous variables were categorized | 7-8 | HAM-D severity bands: no depression 0-7, mild 8-16, moderate 17-23, severe >=24 (Zimmerman et al). DSM-5 Level 1 domains were dichotomised at the thresholds recommended by the American Psychiatric Association. |
|  | 16 | (c) If relevant, consider translating estimates of relative risk into absolute risk for a meaningful time period | - | Not applicable. |
| Other analyses | 17 | Report other analyses done—eg analyses of subgroups and interactions, and sensitivity analyses | 16-17, Appendices 3-4 | Exploratory analyses of the DSM-5 Level 1 domains and all correlation analyses are reported in Multimedia Appendices 3 and 4 and summarised descriptively in the Results. "Analyses of the DSM-5 Level 1 domains and all correlation analyses were not prespecified and are reported as exploratory." Multiple imputation is reported as a sensitivity analysis. |
| **Discussion** |  |  |  |  |
| Key results | 18 | Summarise key results with reference to study objectives | 18 | "depressive symptoms decreased substantially in both the face-to-face and the online follow-up groups... In unadjusted comparisons, HAM-D scores were lower in the online group at the fourth and sixth sessions. However, the groups differed on the primary outcome at baseline, and after adjustment for baseline severity none of these differences remained statistically significant... The most consistent difference between the groups was attendance: participants in the online group attended a higher proportion of scheduled sessions overall." |
| Limitations | 19 | Discuss limitations of the study, taking into account sources of potential bias or imprecision. Discuss both direction and magnitude of any potential bias | 22 | Eleven numbered limitations are given, covering preference-based allocation and residual confounding; the absence of an equivalence design; the absence of blinding; differential attrition and non-random missingness; unrecorded reasons for non-attendance; multiplicity; the absence of a structured diagnostic interview; the sample size relative to the effects observed; unstandardised pharmacotherapy; unstandardised session content; and single-centre recruitment with a predominantly female sample. |
| Interpretation | 20 | Give a cautious overall interpretation of results considering objectives, limitations, multiplicity of analyses, results from similar studies, and other relevant evidence | 18-23 | "These findings do not establish that the two modalities are equivalent. Demonstrating equivalence requires a predefined equivalence margin and confidence-interval-based testing... the absence of a statistically significant difference in an observational study of this size cannot be interpreted as evidence of equivalence. The study is therefore best understood as a descriptive account of the feasibility of online psychiatric follow-up and of its association with continuity of care, rather than as a comparative effectiveness claim." |
| Generalisability | 21 | Discuss the generalisability (external validity) of the study results | 23 | "participants were recruited at a single tertiary university hospital, the sample was predominantly female, and individuals without access to the equipment required for videoconferencing were ineligible, which limits generalizability and reflects a wider concern about digital inequality." |
| **Other information** |  |  |  |  |
| Funding | 22 | Give the source of funding and the role of the funders for the present study and, if applicable, for the original study on which the present article is based | 23 | "The authors declared no financial support was received for this work." |

*Give information separately for exposed and unexposed groups where indicated by an asterisk.*

*Note: An Explanation and Elaboration article discusses each checklist item and gives methodological background and published examples of transparent reporting. The STROBE checklist is best used in conjunction with this article (freely available on the websites of PLoS Medicine, Annals of Internal Medicine, and Epidemiology). Information on the STROBE Initiative is available at www.strobe-statement.org.*
