# Supplementary material for: Symptom Trajectories and Attendance in Face-to-Face and Online Psychiatric Follow-Up for Major Depressive Disorder and Anxiety Symptoms: Prospective Observational Cohort Study
Source: JMIR Form Res. 2026 Oct 19;10:e97656. doi: 10.2196/97656 (PMC13728771; doi:10.2196/97656)
Supplement: Supplementary file 2 [file formative_v10i1e97656_app2.docx]

**Interview duration and setting**

- Initial interview: approximately 60 minutes, conducted in a quiet and private environment, either face to face or by videoconference.
- Follow-up interviews: approximately 30 minutes, conducted at the scheduled date and time.

**Objectives of the interview**

- Assessment of current psychiatric symptoms.
- Monitoring of depressive and anxiety symptom severity.
- Evaluation of treatment adherence and possible medication-related adverse effects.
- Provision of psychoeducation and supportive clinical interventions.
- Identification of situations requiring additional outpatient psychiatric evaluation or treatment modification.

**Interview content**

- Psychiatric symptom assessment conducted in accordance with DSM-5 diagnostic criteria.
- Administration of the scheduled outcome measures: the Hamilton Depression Rating Scale (HAM-D), the Hamilton Anxiety Rating Scale (HAM-A), the DSM-5 Level 2 Depression Scale, and the DSM-5 Level 1 Cross-Cutting Symptom Measure.
- Review of pharmacological treatment, based on patient report, treatment adherence, and monitoring of adverse effects.
- Functional assessment covering daily functioning, interpersonal relationships, and occupational or academic functioning, with individualized behavioral recommendations where appropriate.
- Clinical assessment of suicide risk at every follow-up session.

**Format-specific applications**

- Face-to-face group: all sessions were conducted in the outpatient clinic under conditions ensuring confidentiality and privacy.
- Online group: sessions were conducted by Zoom or WhatsApp video call with the camera on. Audio and video quality were checked before each session began, and a reminder regarding privacy and confidentiality was provided at the start of every session.

**Psychoeducation components**

- Information about the nature of depression, the treatment process, and the risk of relapse.
- Explanation of medication effects and possible adverse effects.
- Guidance on sleep hygiene, stress management, and the maintenance of regular daily routines.
- Delivery of psychoeducation adapted to the consultation format.

**Evaluation and documentation**

- At each session, scale scores, symptom changes, treatment-related clinical observations, and patient-reported concerns were documented systematically.
- Pharmacological treatment decisions were made by the outpatient psychiatrists according to routine clinical practice, independently of the research assessments.

*No structured psychotherapy protocol was delivered. Sessions reflected routine psychiatric outpatient practice, and the timing, structure, and clinical content of sessions were identical in the two groups.*
