# Supplementary material for: Symptom Trajectories and Attendance in Face-to-Face and Online Psychiatric Follow-Up for Major Depressive Disorder and Anxiety Symptoms: Prospective Observational Cohort Study
Source: JMIR Form Res. 2026 Oct 19;10:e97656. doi: 10.2196/97656 (PMC13728771; doi:10.2196/97656)
Supplement: Supplementary file 3 [file formative_v10i1e97656_app3.docx]

| Subscaleᵃ | 0th Session | 1st Session | 2nd Session | 4th Session | 6th Session |
| --- | --- | --- | --- | --- | --- |
| **Depression** |  |  |  |  |  |
| Face-to-Face n (%) | 60 (100.0) | 41 (80.4) | 31 (67.4) | 17 (39.5) | 11 (25.6) |
| Online n (%) | 60 (100.0) | 41 (71.9) | 23 (41.8) | 5 (9.6) | 6 (12.2) |
| Test statisticᵇ (df) / *P* | 0.0 (1) / 1.00 | 1.5 (1) / .22 | 5.9 (1) / .02 | 11.8 (1) / .001 | 3.1 (1) / .078 |
| **Anger** |  |  |  |  |  |
| Face-to-Face n (%) | 50 (83.3) | 17 (33.3) | 17 (37.0) | 8 (18.6) | 8 (18.6) |
| Online n (%) | 47 (78.3) | 18 (31.6) | 19 (34.5) | 12 (23.1) | 8 (16.3) |
| Test statistic (df) / *P* | 0.5 (1) / .49 | 0.1 (1) / .79 | 0.03 (1) / .86 | 0.3 (1) / .60 | 0.2 (1) / .69 |
| **Mania** |  |  |  |  |  |
| Face-to-Face n (%) | 9 (15.0) | 4 (7.8) | 2 (4.3) | 2 (4.7) | 4 (9.3) |
| Online n (%) | 2 (3.3) | 3 (5.3) | 2 (3.6) | 2 (3.8) | 2 (4.1) |
| Test statistic (df) / *P* | 4.9 (1) / .03 | Fisher / .70 | Fisher / 1.00 | Fisher / 1.00 | Fisher / .41 |
| **Anxiety** |  |  |  |  |  |
| Face-to-Face n (%) | 54 (90.0) | 36 (70.6) | 32 (69.6) | 17 (39.5) | 12 (27.9) |
| Online n (%) | 54 (90.0) | 28 (49.1) | 24 (43.6) | 15 (28.8) | 9 (18.4) |
| Test statistic (df) / *P* | 0.0 (1) / 1.00 | 5.8 (1) / .02 | 6.1 (1) / .013 | 1.2 (1) / .27 | 1.8 (1) / .22 |
| **Somatic Symptoms** |  |  |  |  |  |
| Face-to-Face n (%) | 44 (73.3) | 24 (47.1) | 26 (56.5) | 13 (30.2) | 14 (32.6) |
| Online n (%) | 45 (75.0) | 33 (57.9) | 23 (41.8) | 13 (25.0) | 10 (20.4) |
| Test statistic (df) / *P* | 0.04 (1) / .86 | 1.1 (1) / .31 | 1.9 (1) / .17 | 0.3 (1) / .57 | 2.2 (1) / .14 |
| **Suicidal Ideation** |  |  |  |  |  |
| Face-to-Face n (%) | 8 (13.3) | 6 (11.8) | 2 (4.3) | 5 (11.6) | 3 (7.0) |
| Online n (%) | 13 (21.7) | 0 (0.0) | 0 (0.0) | 4 (7.7) | 3 (6.1) |
| Test statistic (df) / *P* | 1.4 (1) / .23 | Fisher / .009 | Fisher / .21 | Fisher / .73 | Fisher / 1.00 |
| **Psychosis** |  |  |  |  |  |
| Face-to-Face n (%) | 14 (23.3) | 6 (11.8) | 3 (6.5) | 4 (9.3) | 0 (0.0) |
| Online n (%) | 17 (28.3) | 7 (12.3) | 3 (5.5) | 4 (7.7) | 4 (8.2) |
| Test statistic (df) / *P* | 0.4 (1) / .53 | 0.0 (1) / .97 | Fisher / 1.00 | Fisher / 1.00 | Fisher / .12 |
| **Sleep Problems** |  |  |  |  |  |
| Face-to-Face n (%) | 36 (60.0) | 25 (49.0) | 19 (41.3) | 7 (16.3) | 3 (7.0) |
| Online n (%) | 40 (66.7) | 23 (40.4) | 15 (27.3) | 8 (15.4) | 5 (10.2) |
| Test statistic (df) / *P* | 0.6 (1) / .45 | 1.0 (1) / .32 | 2.0 (1) / .16 | 0.01 (1) / .91 | Fisher / .72 |
| **Memory** |  |  |  |  |  |
| Face-to-Face n (%) | 22 (36.7) | 18 (35.3) | 13 (28.3) | 6 (14.0) | 6 (14.0) |
| Online n (%) | 30 (50.0) | 32 (56.1) | 26 (47.3) | 13 (25.0) | 8 (16.3) |
| Test statistic (df) / *P* | 2.2 (1) / .14 | 4.3 (1) / .04 | 4.1 (1) / .042 | 1.8 (1) / .18 | 0.1 (1) / .79 |
| **Repetitive Thoughts/Behaviors** |  |  |  |  |  |
| Face-to-Face n (%) | 38 (63.3) | 22 (43.1) | 13 (28.3) | 7 (16.3) | 10 (23.3) |
| Online n (%) | 34 (56.7) | 29 (50.9) | 29 (52.7) | 16 (30.8) | 11 (22.4) |
| Test statistic (df) / *P* | 0.6 (1) / .46 | 0.5 (1) / .48 | 6.6 (1) / .01 | 2.7 (1) / .10 | 0.1 (1) / .83 |
| **Dissociation** |  |  |  |  |  |
| Face-to-Face n (%) | 36 (60.0) | 18 (35.3) | 15 (32.6) | 8 (18.6) | 9 (20.9) |
| Online n (%) | 32 (53.3) | 11 (19.3) | 8 (14.5) | 2 (3.8) | 2 (4.1) |
| Test statistic (df) / *P* | 0.5 (1) / .46 | 3.8 (1) / .052 | 4.4 (1) / .04 | Fisher / .04 | Fisher / .01 |
| **Personality Functioning** |  |  |  |  |  |
| Face-to-Face n (%) | 47 (78.3) | 24 (47.1) | 25 (54.3) | 15 (34.9) | 13 (30.2) |
| Online n (%) | 45 (75.0) | 17 (29.8) | 8 (14.5) | 11 (21.2) | 8 (16.3) |
| Test statistic (df) / *P* | 0.2 (1) / .67 | 3.7 (1) / .05 | 17.3 (1) / <.001 | 2.2 (1) / .14 | 3.0 (1) / .09 |
| **Substance Use** |  |  |  |  |  |
| Face-to-Face n (%) | 25 (41.7) | 19 (37.3) | 14 (30.4) | 12 (27.9) | 11 (25.6) |
| Online n (%) | 27 (45.0) | 25 (43.9) | 31 (56.4) | 21 (40.4) | 19 (38.8) |
| Test statistic (df) / *P* | 0.1 (1) / .71 | 0.4 (1) / .54 | 7.3 (1) / .007 | 1.6 (1) / .20 | 1.4 (1) / .23 |

*a Subscales were dichotomized as above versus below the predefined cutoff values.*

*b Pearson chi-square test (df=1) was used for comparisons when test assumptions were met; otherwise, Fisher’s exact test was applied when expected cell counts were <5. Percentages were calculated based on the number of participants available at each session.*
