# Supplementary material for: Symptom Trajectories and Attendance in Face-to-Face and Online Psychiatric Follow-Up for Major Depressive Disorder and Anxiety Symptoms: Prospective Observational Cohort Study
Source: JMIR Form Res. 2026 Oct 19;10:e97656. doi: 10.2196/97656 (PMC13728771; doi:10.2196/97656)
Supplement: Supplementary file 4 [file formative_v10i1e97656_app4.docx]

**A. HAM-D**

| Variable | 0th Session (Baseline) | 1st session | 2nd session | 4th session | 6th session |
| --- | --- | --- | --- | --- | --- |
| Sex |  |  |  |  |  |
| ρᵃ | −0.15 | −0.24 | −0.24 | −0.31 | −0.33 |
| P value | .11 | .01 | .02 | .003 | <.001 |
| Marital status |  |  |  |  |  |
| ρᵇ | −0.09 | −0.07 | −0.12 | −0.23 | −0.38 |
| P value | .33 | .45 | .25 | .02 | <.001 |
| Number of children |  |  |  |  |  |
| ρc | 0.10 | 0.09 | 0.01 | 0.17 | 0.25 |
| P value | .27 | .38 | .89 | .10 | .02 |

*ᵃ Sex was coded as 1=female and 2=male.*

*ᵇ Marital status was coded as 1=married and 2=single.*

*ᶜ Number of children was analyzed as a continuous variable.*

**B. HAM-A**

| Variable | 0th Session (Baseline) | 1st session | 2nd session | 4th session | 6th session |
| --- | --- | --- | --- | --- | --- |
| Marital status |  |  |  |  |  |
| ρᵃ | −0.15 | −0.02 | −0.20 | −0.27 | −0.32 |
| P value | .25 | .87 | .18 | .08 | .04 |
| Physical illness |  |  |  |  |  |
| ρᵇ | −0.26 | −0.27 | −0.38 | −0.28 | −0.27 |
| P value | .046 | .06 | .009 | .07 | .09 |
| Psychiatric history |  |  |  |  |  |
| ρc | 0.01 | −0.32 | −0.23 | −0.31 | −0.07 |
| P value | .933 | .02 | .13 | .04 | .67 |
| Alcohol use |  |  |  |  |  |
| ρd | −0.01 | 0.00 | −0.23 | 0.09 | −0.32 |
| P value | .95 | 1.00 | .12 | .57 | .04 |
| Smoking |  |  |  |  |  |
| ρe | −0.14 | −0.03 | −0.23 | −0.08 | −0.39 |
| P value | .28 | .82 | .12 | .60 | .01 |

*ᵃ Marital status was coded as 1=married and 2=single.*

*ᵇ Physical illness was coded as 1=no and 2=yes.*

*ᶜ Psychiatric history was coded as 1=no and 2=yes.*

*ᵈ Alcohol use was coded as 1=no and 2=yes.*

*ᵉ Smoking was coded as 1=no and 2=yes.*
